# Parental Knowledge, Attitude and Practices Toward Cariogenic Potential of Pediatric Oral Medications

**DOI:** 10.3390/children12081100

**Published:** 2025-08-21

**Authors:** Reham M. Al-Amoudi, Heba Mohamed Elkhodary, Shahad N. Abudawood, Azza El-Housseiny, Osama M. Felemban

**Affiliations:** 1 Pediatric Dentistry Department, Taif Dental Specialty Center, Ministry of Health, Taif 26514, Saudi Arabia; realamoudi@moh.gov.sa; 2 Pediatric Dentistry Department, Faculty of Dentistry, King Abdulaziz University, P.O. Box 80200, Jeddah 21589, Saudi Arabia; hkhodary@kau.edu.sa (H.M.E.); omfelemban@kau.edu.sa (O.M.F.); 3 Department of Pedodontics and Oral Health, Faculty of Dental Medicine for Girls, Al Azhar University, Cairo 11651, Egypt; 4 Pediatric Dentistry Department, Faculty of Dentistry, Alexandria University, Alexandria 21526, Egypt; azza.elhousseiny@dent.alex.edu.eg

**Keywords:** pediatric dentistry, cariogenic pediatric medications, dental caries, dental erosion

## Abstract

**Background/Objectives**: Sugar added to pediatric oral medications may increase the risk of dental caries and erosion. Parental awareness and practices play a crucial role in minimizing the oral health risks associated with such medications. Therefore, the aim was to assess parents’ knowledge, attitudes, and practices regarding the cariogenic potential of pediatric oral medications in the Kingdom of Saudi Arabia. **Methods**: A cross-sectional study was conducted using an online questionnaire. Parents who had at least one child between 2 and 12 years old were included. Participants were categorized into three groups based on the long-term intake of medications and their child’s health status. Group 1: Parents of children with chronic diseases on long-term medications. Group 2: Parents of healthy children on long-term medications. Group 3: Parents of healthy children not on long-term medications. **Results**: A total of 2195 responses were collected. The majority of parents were aged 31–40 years. More than half of the mothers and fathers had a diploma or a university degree (59% and 54.3%, respectively). Although a high proportion of the parents (78–83%) were aware that medications often contained sugar, only 46–50% were aware of their cariogenic potential, while 27–38% speculated about their erosive potential. In terms of practices, most parents encouraged their children to drink water (70–71%) or rinse their mouths (14–20%) after medication intake. **Conclusions**: While most parents were aware that pediatric oral medications may contain sugar, there was a lack of proactive measures to mitigate their negative impact on oral health. Further studies are still needed to evaluate and improve public awareness and practices regarding the oral health risks associated with pediatric medications.

## 1. Introduction

Oral health is a crucial component of children’s overall growth and development. Globally, the prevalence rates of dental caries in primary and permanent teeth among children vary across different continents, with approximately 65.6% of school-going Saudi children affected [1]. Dental caries is a complex and multi-factorial chronic disease that is formed over time, primarily due to decreases in the salivary pH due to acids produced by plaque organisms, primarily *Streptococcus mutans*, in the presence of fermentable sugars, resulting in enamel demineralization [2].

Children are frequently prescribed medications for various health problems. Oral administration is the most common route, with different forms available, such as syrups, capsules, suspensions, solutions, tablets, and chewables [3,4]. The liquid/syrup form is frequently prescribed because it is well-absorbed, convenient for administration, and well-tolerated by young children [3,5,6]. Manufacturers often add inert ingredients like flavoring, coloring agents, and high concentrations of fermentable sugar in the form of sucrose, fructose, or glucose as taste masking, improving compliance among children and ease of swallowing [7]. Sucrose is the main sugar added to pediatric medications, whereas glucose is used at lower levels, while fructose, lactose, and maltose are rarely encountered in such formulations. Sucrose is known to have the most cariogenic potential, whereas glucose, fructose, and maltose pose a moderate risk, and lactose poses the lowest cariogenic potential among these sugars [8]. Studies have reported that sucrose concentrations in pediatric liquid medications can range from 3.7% to 67.0% by weight, which is higher than the sugar content found in ice cream (15.1%) and soft drinks (4.3%) [9,10]. Additionally, glucose concentrations in these medications have been found to vary between 2.10% and 40.19% [11]. The frequent use of such sweetened medications, especially without proper oral hygiene practices, may increase the risk of dental caries and erosion in children [12]. Other factors that increase the cariogenic and erosive potential of pediatric oral medications (POMs) include the sugar concentration, viscosity, pH, frequency, the duration of usage, and the time of administration [13]. The pH of commonly used pediatric medications, such as antibiotics, analgesics, antipyretics, cough and cold medications, bronchodilators, and anticonvulsants, has been reported to be low and acidic [14]. Some POMs are comparable to sweets with low pH values, thus increasing the risk of dental erosion [15]. Reported pH values of these medications range from 3.40 to 5.38, levels sufficient to cause a reduction in enamel surface microhardness, increased roughness, demineralization, and, with prolonged use, dental caries and erosion [10,15,16,17]. It is important to note that dental erosion in primary teeth is commonly observed and influenced by multiple factors, with dry mouth induced by certain medications as a notable contributor to its development and progression [18]. Furthermore, the time of administration is an important factor as oral clearance and salivary flow are lowered during sleep [19].

Parents are typically responsible for administering medications to their children. However, most parents are unaware of the hidden sugars and the cariogenic potential of these medications, as their primary concern is addressing their child’s immediate health issues rather than considering the long-term side effects on oral health [13,20,21]. To our knowledge, no previous studies have assessed parental understanding of the impact of pediatric medications on children’s dental health in Saudi Arabia. Therefore, the aim of this study was to evaluate parental knowledge, attitude, and practices (KAP) regarding the cariogenic and erosive potential of pediatric oral medications among parents of three groups of children, including medically compromised children on long-term medications, healthy children on long-term medications, and healthy children not on long-term medications. In addition, the study sought to assess the influence of parental and familial demographics on parental KAP regarding the cariogenic and erosive potential of pediatric oral medications.

## 2. Materials and Methods

### 2.1. Study Design and Setting

This was a cross-sectional study conducted using an online survey over a period of 6 weeks, from 14 December 2023 to 31 January 2024. This study was reported according to the standards of Strengthening the Reporting of Observational Studies in Epidemiology (STROBE) [22]. Parents who agreed to participate in the study were required to approve a consent form, which briefly explained the objectives of the study. Participants were informed of the voluntary nature of their involvement and the confidentiality of their personal information. The study findings were accessible only to the research team and were used solely for research purposes. None of the parents was permitted to begin the online survey questionnaire without first providing consent to participate. This research was approved by the Research Ethics Committee of the Faculty of Dentistry at King Abdulaziz University KAU, Jeddah, KSA (209-01-21).

### 2.2. Participants and Sampling Methods

The snowball sampling technique was used to include the study participants and distribute the questionnaire through WhatsApp during the period from 14 December 2023 to 31 January 2024. The questionnaire was shared in several WhatsApp groups of the researchers as a Google Form link. Group members were requested to forward the questionnaire to their contacts. Additionally, patients and their companions in the outpatient clinic waiting areas of King Abdulaziz University Hospital (KAUH) and King Abdulaziz University Dental Hospital (KAUDH) were approached to be included in the study, and they were instructed to share the questionnaire with their family and friends through their WhatsApp groups. This snowball sampling technique facilitated non-probability sampling across various regions of Saudi Arabia. The intention was to collect a large enough sample size to appropriately represent the target group (parents living in Saudi Arabia) within a reasonable timeframe. Participants were required to have at least one child aged between 2 and 12 years and to be able to read and understand Arabic. Questionnaires that were incomplete or filled out by family members other than the parents were excluded from the study. The study sample was divided into three groups based on the health status of the child and the use of long-term medications. The three groups consisted of Group 1: parents of children with chronic diseases who were on long-term medications. Group 2: parents of healthy children on long-term medications. Group 3: parents of healthy children not on long-term medications (Group 3).

### 2.3. The Questionnaire

To ensure content validity, the survey, consisting of 31 questions, was reviewed by nine experts (six pediatric dentists and three dental public health faculty members) at KAU. The experts evaluated the items based on their relevance to the study, clarity, simplicity, and ambiguity. The experts were asked to evaluate each item on a 4-point scale. The scale indicated how favorable the question was by selecting the numbers 1 to 4, where 4 corresponded to the most favorable criteria, while 1 was the least favorable. These ratings were further statistically analyzed to calculate the validity of the questionnaire. The Content Validity Index (CVI) was found to be excellent (98.39%) using the Average method and very good (78.13%) using the Universal method of calculation based on the method introduced by Zamanzadeh [23]. To ensure the reliability of the questionnaire and decrease random errors, test re-test reliability was evaluated. A sample of thirty randomly selected parents who had children between 2 and 12 years old were invited to participate in a pilot test. Two weeks later, the questionnaire was re-sent to the same participants, and the responses were compared statistically. The value of the weighted kappa used to evaluate test re-test reliability was 0.96 (95% CI, 0.94–0.98), which corresponded to an almost perfect agreement, as suggested by Landis and Koch [24].

The questionnaire consisted of a total of thirty-one questions divided into four parts. The first part focused on the demographic information and socioeconomic status of the parents, including age, marital status, parents’ educational level, monthly household income, number of children in the household 18 years of age or younger, whether the child was living with his or her mother or father or both, and homeownership status (rented or owned). The subsequent sections included questions on the parents’ KAP regarding the cariogenic and erosive potential of pediatric medications and their impact on oral health (Appendix A).

### 2.4. Statistical Methods

Descriptive statistics were computed and represented in the form of frequencies and percentages. The Chi-square test was used to compare the groups’ questionnaire answers and then to examine the association between demographic characteristics and key questionnaire items. A total score of correct responses was computed for each participant, and then the scores were dichotomized based on the median. Participants who scored in the lower half of the range were classified as having a low KAP, while those who scored in the upper half were classified as having a high KAP. Multiple logistic regression analysis was utilized to model the effects of demographics and socioeconomic status on the dichotomous KAP score (dependent variable) while accounting for confounding variables. The level of significance was set at 0.05. The Statistical Package for Social Sciences (SPSS) software was used to analyze the data (IBM SPSS Statistics for Windows, Version 20.0, Armonk, NY, USA, IBM Corp.).

## 3. Results

**Figure 1:**
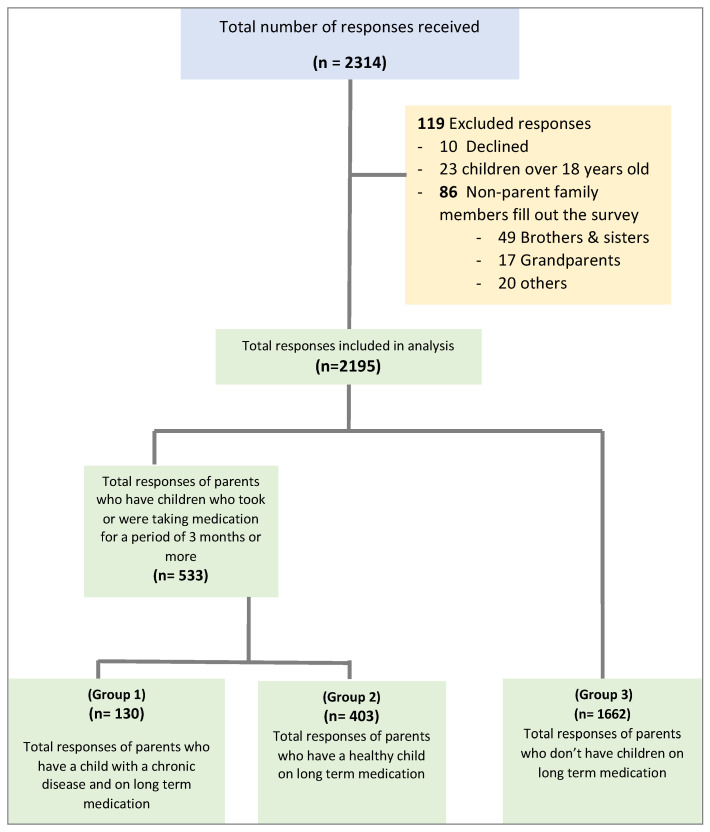
Flowchart of data collection process.

A total of 2314 answers were received. Figure 1 depicts the number of participants in the study. Table 1. Compares the sociodemographic parameters of individuals. Group 1 mothers and fathers (parents of children with chronic diseases on long-term drugs) were more likely to be older (41 years or more) than Groups 2 and 3 (*p* < 0.001 and *p* < 0.001, respectively). In terms of parental education, mothers and fathers in Group 2 (parents of healthy children on long-term medications) were more likely to have higher educational degrees (e.g., master’s or PhD) than mothers and fathers in Groups 1 and 3 (*p* < 0.001 and *p* < 0.001, respectively). When compared to Groups 1 and 2, participants in Group 3 (parents of healthy children who are not on long-term drugs) were more likely to be in the middle-income level (10,001–20,000 SAR). Approximately one-third of Group 1 participants (31.5%) had four or more children under the age of 18, compared to 14.4% of Group 2 and 18.8% of Group 3 (*p* < 0.001).

Questions about drug administration habits were asked to better investigate the practices of the groups that had children on long-term medications (Group 1: parents of children with chronic diseases on long-term medications and Group 2: parents of healthy children on long-term medications), and the results are shown in Table 2. Group 1 participants were more likely to use syrup drugs (*p* = 0.002), followed by tablets (*p* < 0.001), and inhalers (*p* < 0.001) when compared to Group 2 participants. On the other hand, Group 2 participants were more likely to consume chewable medications than Group 1 participants (*p* < 0.001). Group 1 participants were more likely (65.4%) to have taken the drugs for a longer period of time (>12 months) than Group 2 participants (18.6%), and the difference was statistically significant (*p* < 0.001). In addition, compared to Group 2, Group 1 participants were more likely to take their prescriptions in the morning (*p* = 0.005), evening (*p* < 0.001), or at bedtime (*p* < 0.001). Group 1 children were more likely (70.3%) to take their prescriptions on a daily basis than Group 2 children (40.8%), and the difference was statistically significant (*p* < 0.001).

Table 3 compares the knowledge, attitudes, and practices of the parents in the three groups regarding the cariogenic and erosive risk of pediatric medicines. Parents were asked if children’s oral medications led to dry mouth. Parents of children in Group 1 were more likely to consider this to be true than parents of children in Groups 2 and 3 (45.4% vs. 38.5% and 34.0%, respectively), and this difference was statistically significant (*p* = 0.013). Parents were questioned about whether children’s oral medications caused tooth erosion. The majority of parents lacked knowledge on whether these medications can cause erosion, with Group 3 having the highest percentage (19.2% answered No and 53.1% did not know) compared to Group 2 (15.6% answered No and 51.4% did not know) and Group 1 (16.9% answered No and 44.6% did not know), and these differences were statistically significant (*p* = 0.024). The importance of drinking water or rinsing one’s mouth after taking an oral medication was also asked of the parents. Group 1 had a higher percentage of respondents who agreed with the statement (83.1%) than did Group 2 (73.7%) and Group 3 (71.8%), but the difference was not statistically significant (*p* = 0.062). Also, the parents were asked if they had read the ingredients on the packet or in the information sheet inside the box before using the medication. While Group 2 (63.3%) and Group 3 (57.8%) were less likely to read the ingredients, the difference was statistically significant (*p* = 0.018), with more than two-thirds of Group 1 stating that they did so (67.7%). After giving the oral medications, parents were asked if they typically ask their children to drink water, rinse their mouths, brush their teeth, or do nothing. Compared to (85.4%) parents in Group 2 and (85.7%) in Group 3, the majority of parents in Group 1 (93.8%) asked their kids to drink water (*p* = 0.032).

Overall, in the knowledge, attitude, and behavior questions, Groups 1 and 2 performed preferentially better (4.58 ± 1.69 and 4.28 ± 1.58, respectively) than Group 3 (4.04 ± 1.63), and the difference was statistically significant (*p* < 0.001). When the overall score was dichotomized into high vs. low KAP, greater percentages of Group 1 participants (74.6%) performed notably better compared to percentages of participants in Group 2 (70.7%) and Group 3 (64.3%) in terms of knowledge, attitude, and behavior scores, and the difference was statistically significant (*p* = 0.005).

An adjusted logistic regression model was used to assess the associations between the groups and the dichotomized KAP score (dependent variable) while taking into account potential confounding factors. Even after adjusting for other variables, Groups 1 and 2 had significantly higher probabilities of having high KAP scores than Group 3 (OR = 1.60, 95% CI 1.06–2.42, *p* = 0.026; OR = 1.35, 95% CI 1.06–1.72, *p* = 0.014). Families with younger fathers or mothers with lower levels of education also had a significantly lower likelihood of having high KAP scores and the results are shown in Table 4.

## 4. Discussion

This study was conducted as a non-experimental cross-sectional study with the objective of assessing parents’ current knowledge, attitudes, and practices (KAP) regarding the cariogenic and erosive potential of pediatric oral medications (POMs) among various groups of children and to evaluate the family sociodemographic influence on their KAP. The results of this study show several findings. Medically compromised children on long-term medications used different forms of POMs for longer durations and took their medications on a daily basis at various times. Additionally, most of the parents of children in this sample had good knowledge, attitudes, and practices regarding pediatric oral medications. However, differences in the KAP were noted between the groups. In addition to having a medically compromised or healthy child on long-term medication, the father’s age and the mother’s education level were significant factors/variables associated with the parental KAP score.

In terms of parents’ knowledge of the sugar content of POMs, no statistically significant difference between groups was evident, but it is worth noting that more than two-thirds of participants were aware that POMs contained sugar. This was similar to the findings of previous studies, in which parents were mostly unaware of a connection between medications and the development of dental caries [20,21]. However, this disagrees with the findings of Anantharaj et al. and Mathew et al., who reported that parents and pediatricians may lack awareness of the hidden sugar content of POMs, as well as their deleterious effects on children’s oral health [3,25]. Furthermore, while many parents in the current study were aware that their children’s medications contained sugar, fewer parents were aware that sugar-containing POMs might cause dental caries. This was in accordance with the findings of a previous study published by Thosar et al., which found that mothers were mostly unaware of the link between regular POM usage and dental caries, and few believed that these medications could not cause dental caries [26].

The present study demonstrated significant differences in the medication form, duration, timing, and frequency of administration between parents of children on long-term medications in Group 1 and Group 2. It is important to note that this information was collected for descriptive and comparative purposes only. The study did not assess specific disease types, diagnoses, or therapeutic drug classes, which may significantly influence the necessity, formulation, or substitutability of medications. These clinical factors could have affected parental practices and attitudes, particularly in cases involving high-morbidity conditions requiring complex or irreplaceable pharmacotherapy. More specifically, children in Group 1 (medically compromised on long-term medications) were reported to use a broader range of medication forms, including syrups, tablets, chewables, and inhalers administered daily at various times and for longer durations. In contrast, children in Group 2 (healthy but on long-term medications) primarily used syrups, followed by chewable tablets. The European Academy of Pediatric Dentistry advises against the nighttime intake of sugar-containing beverages, given their established role in caries development [27]. Although there are currently no specific guidelines regarding the timing of POMs administration, minimizing intake before bedtime is recommended to reduce the cariogenic effect.

Moreover, the study findings showed that the majority of parents, particularly those of healthy children not on long-term medications (Group 3), were unaware of the potential side effects of POMs, such as dental erosion. In contrast, parents of medically compromised children on long-term medications demonstrated greater awareness of these side effects compared to parents of healthy children, regardless of their medication use. One possible explanation for this discrepancy is that parents may associate the sweet taste of medications with the presence of sugars but may not recognize the role of acids in the formulation. In recent years, non-carious dental conditions, such as tooth erosion, have become increasingly prevalent, particularly in industrialized nations, with prolonged and frequent contact of low-pH medications contributing to their development or accelerating their progression [28]. The reported prevalence of erosive tooth wear among children varies significantly, ranging from 5.7% to 78%, and no recent reviews have evaluated the contributing factors [29]. Furthermore, research investigating parental awareness of drug-induced xerostomia and erosion to POMs use remains limited.

The present study found that most parents recognized the importance of drinking water, rinsing, or brushing following the intake of POMs. Among them, 93.8% of parents of medically compromised children on long-term medications in Group 1 encouraged this practice, compared to 85.4% in Group 2 and 85.7% in Group 3. These findings are consistent with previous research, which reported similar parental behaviors but noted a lack of additional oral hygiene instructions [3,26]. Additionally, more than half of the respondents in each group reported reading medication labels or package inserts before use, with 67.7% in Group 1, 63.3% in Group 2, and 57.8% in Group 3, confirming this practice. These findings are comparable to those by Eymirli et al., where 71.4% of parents read labels and 88.4% reviewed package inserts. Variations in findings may be attributed to differences in sample size, grouping methods, and the proportion of children with chronic illnesses requiring ongoing medication [30].

Regarding the overall parental KAP in the current study, parents of medically compromised children in Group 1 demonstrated greater awareness of the importance of oral health and the potential effects of medications compared to the other study groups. This agrees with Shah et al., who found that caregivers of special health needs patients generally possessed sufficient knowledge but exhibited weaker attitudes toward oral health [31]. This may be because such parents may prioritize their child’s medical condition, potentially leading to the neglect of long-term oral health consequences associated with medication use.

Moreover, the logistic regression model found a significant association between the group classification, fathers’ age, mothers’ educational level, and their KAP regarding oral medications with respect to their child. Parents of medically compromised children on long-term medications (Group 1) and parents of healthy children on long-term medications (Group 2) had higher odds of achieving higher KAP scores in relation to parents of healthy children who are not on long-term medications (Group 3). This could be due to greater exposure to medical information as these parents often have more frequent interactions with healthcare providers, such as pediatricians, pharmacists, and specialists, who provide guidance on medication use, side effects, and management strategies. In contrast, parents of healthy children may not have the same level of exposure to medical information, potentially leading to less awareness and knowledge about medication management [32]. Furthermore, families with younger fathers (<40 years) as well as mothers with lower levels of education were significantly less likely to exhibit high KAP scores. Younger parents may have lower KAP due to limited experience, while older parents might have better awareness and more established health practices. Upon reviewing the available literature, there is a scarcity of studies specifically examining the relationship between a father’s age and their KAP concerning the cariogenic and erosive potential of pediatric oral medications. Our findings are in agreement with existing literature that emphasizes the influence of maternal education on KAP related to oral health. Higher levels of maternal education are consistently associated with better oral hygiene practices and lower incidence of dental caries in children [33,34].

This study has several strengths and limitations. Among its strengths are the relatively large sample size and the inclusion of participants from different regions, which adds diversity to the responses. Additionally, the questionnaire demonstrated good validity and test-retest reliability, which supports the credibility of the findings. However, certain limitations must be acknowledged. The study employed a non-probability convenience snowball sampling technique, which limits the generalizability of results, as not all individuals had an equal chance of being included. While WhatsApp distribution allowed a broad reach within Saudi Arabia, it may not fully represent the entire population. Furthermore, our sample might be skewed toward families of higher socioeconomic status because the survey was distributed online. Online surveys are challenging to access for low-income parents who lack smartphones. The anonymous nature of the survey also raised the possibility of multiple response bias (e.g., duplicate responses or both parents responding for the same child), which could not be controlled. Furthermore, self-reporting and recall bias may have led participants to over-report positive behaviors. Future research should aim to use random or stratified sampling methods to enhance representativeness and consider integrating clinical oral health data alongside parental reports. Moreover, studies focusing on specific disease categories and types of medications could offer more detailed insights into how medical conditions influence parental knowledge, attitudes, and practices.

## 5. Conclusions

Based on the findings of the current study, it can be concluded that parents generally had relatively fair awareness of the high sugar content in pediatric oral medications; however, their knowledge regarding the potential consequences of such medications—such as dental decay, dry mouth, and dental erosion—was limited. Parental KAP regarding the cariogenic potential of pediatric oral medications was higher among parents of medically compromised children on long-term medications than among parents of healthy children, regardless of whether those children were taking long-term medications. Additionally, mothers’ education levels and fathers’ ages showed significant associations with parents’ KAP related to the cariogenic potential of pediatric medications. These results highlight the need for targeted educational efforts and support for parents, particularly those with limited exposure to healthcare systems or lower educational backgrounds, to mitigate the oral health risks associated with the long-term use of pediatric medications. Healthcare providers should actively educate parents, particularly those caring for children with special healthcare needs, about the sugar content in oral medications and their potential harmful effects on dental health. Public health campaigns play a vital role in raising awareness and providing guidance on managing potential side effects of POMs. This approach can aid in prevention, early detection, and management, thereby mitigating associated oral health issues.

## Figures and Tables

**Table 1:** Sociodemographic characteristics of the study participants.

Characteristics	Responses	Group 1 (n = 130)	Group 2 (n = 403)	Group 3 (n = 1662)	*p*-Value
Mother age	≤30 years	15 (11.5)	112 (27.8)	383 (23.0)	<0.001 *
	31–40 years	63 (48.5)	213 (52.9)	884 (53.2)	
	41 years or more	52 (40.0)	78 (19.4)	395 (23.8)	
Father age	≤40 years	63 (48.5)	261 (64.8)	898 (54.0)	<0.001 *
	41 years or more	67 (51.5)	142 (35.2)	764 (46.0)	
Mother education	High school or less	30 (23.1)	47 (11.7)	312 (18.8)	<0.001 *
	Diploma or University	69 (53.1)	235 (58.3)	992 (59.7)	
	Higher Education	31 (23.8)	121 (30.0)	358 (21.5)	
Father education	High school or less	35 (26.9)	48 (11.9)	366 (22.0)	<0.001 *
	Diploma or University	63 (48.5)	245 (60.8)	884 (53.2)	
	Higher Education	32 (24.6)	110 (27.3)	412 (24.8)	
Job status of the mother	Employed	46 (35.4)	159 (39.5)	628 (37.8)	0.066
	Owns a business	12 (9.2)	25 (6.2)	83 (5.0)	
	Housewife/ unemployed	69 (53.1)	207 (51.4)	888 (53.4)	
	Retired	2 (1.5)	3 (0.7)	45 (2.7)	
	Other (mainly students)	1 (0.8)	9 (2.2)	18 (1.1)	
Job status of the father	Employed	88 (67.7)	318 (78.9)	1257 (75.6)	0.460
	Owns a business	24 (18.5)	51 (12.7)	249 (15.0)	
	Unemployed	13 (10.0)	24 (6.0)	118 (7.1)	
	Retired	4 (3.1)	8 (2.0)	29 (1.7)	
	Other (mainly students)	1 (0.8)	2 (0.5)	9 (0.5)	
Monthly household income	<10,000 SAR	50 (38.5)	154 (38.2)	604 (36.3)	0.013 *
	10,001–20,000 SAR	39 (30.0)	140 (34.7)	682 (41.0)	
	>20,000 SAR	41 (31.5)	109 (27.0)	376 (22.6)	
Housing	Rented apartment	48 (36.9)	174 (43.2)	690 (41.5)	0.058
	Owned apartment	25 (19.2)	101 (25.1)	467 (28.1)	
	Rented house	10 (7.7)	19 (4.7)	62 (3.7)	
	Owned house	41 (31.5)	94 (23.3)	396 (23.8)	
	Others	6 (4.6)	15 (3.7)	47 (2.8)	
The number of children less than 18 years in the house	1	18 (13.8)	90 (22.3)	329 (19.8)	<0.001 *
	2–3	71 (54.6)	255 (63.3)	1020 (61.4)	
	4 or more	41 (31.5)	58 (14.4)	313 (18.8)	

Group 1: parents of children with chronic diseases on long-term medications. Group 2: parents of healthy children on long-term medications. Group 3: parents of healthy children who are not on long-term medications. * Statistically significant at 0.05 level (chi-square test).

**Table 2:** Responses of parents with children on long-term medications regarding the use of medications for a selected child.

Questions	Responses	Group 1 (n = 130)	Group 2 (n = 403)	*p*-Value
What kind of medications your child uses? (You can choose more than one answer)	Syrup	68 (52.3)	271 (67.6)	0.002 *
	Tablets	45 (34.6)	42 (10.5)	<0.001 *
	Chewing	22 (16.9)	132 (32.9)	<0.001 *
	Inhaler	43 (33.1)	37 (9.2)	<0.001 *
	Other	8 (6.2)	19 (4.7)	0.523
For how long did the child take the medicine?	3–6 months	38 (29.2)	299 (74.2)	<0.001 *
	6–12 months	7 (5.4)	29 (7.2)	
	>12 months	85 (65.4)	75 (18.6)	
How frequent is your child given medications?	When needed	34 (26.6)	222 (55.2)	<0.001 *
	Daily	90 (70.3)	164 (40.8)	
	Weekly or monthly	4 (3.1)	16 (4.0)	
When does/did the child take the medication? (You can choose more than one answer)	Morning	64 (49.2)	142 (35.3)	0.005 *
	After meals	27 (20.8)	182 (45.3)	<0.001 *
	Evening	53 (40.8)	87 (21.6)	<0.001 *
	Bedtime	41 (31.5)	52 (12.9)	<0.001 *
	Other	9 (6.9)	41 (10.2)	0.266

Group 1 = parents of children with chronic diseases on long-term medications. Group 2 = parents of healthy children on long-term medications. * Statistically significant at 0.05 level (chi-square test).

**Table 3:** The knowledge, attitude, and practices toward the cariogenic potential of pediatric medications among parents of healthy children, healthy children on long-term medications, and medically compromised children on long-term medications.

Statements	Responses	Group 1 (n = 130)	Group 2 (n = 403)	Group 3 (n = 1662)	*p*-Value
Children’s oral medications or some of them contain sugar flavorings	True (Correct)	102 (78.5)	338 (83.9)	1339 (80.6)	0.135
	False	3 (2.3)	7 (1.7)	65 (3.9)	
	IDK	25 (19.2)	58 (14.4)	528 (15.5)	
Do some children’s oral medications increase the possibility of tooth decay?	Yes (Correct)	66 (50.8)	201 (49.9)	766 (46.1)	0.415
	No	22 (16.9)	54 (13.4)	254 (15.3)	
	IDK	42 (32.3)	148 (36.7)	642 (38.6)	
Some children’s oral medications lead to dry mouth.	True (Correct)	59 (45.4)	155 (38.5)	565 (34.0)	0.046 *
	False	13 (10.0)	44 (10.9)	224 (13.5)	
	IDK	58 (44.6)	204 (50.6)	873 (52.5)	
Do some children’s oral medications lead to tooth erosion?	Yes (Correct)	50 (38.5)	133 (33.0)	460 (27.7)	0.024 *
	No	22 (16.9)	63 (15.6)	319 (19.2)	
	IDK	58 (44.6)	207 (51.4)	883 (53.1)	
I think it is important to drink water or rinse the mouth after taking an oral medication	Agree (Preferable)	108 (83.1)	297 (73.7)	1194 (71.8)	0.062
	Disagree	5 (3.8)	14 (3.5)	76 (4.6)	
	IDK	17 (13.1)	92 (22.8)	392 (23.6)	
Usually, after giving one of your children medicines orally, you ask him/her to:	Drink water, rinse, or brush (Desirable)	122 (93.8)	344 (85.4)	1425 (85.7)	0.032 *
	Do nothing	8 (6.2)	59 (14.6)	237 (14.3)	
Do you read the ingredients on the outside packet or the attached sheet inside the box before using any medications?	Yes (Desirable)	88 (67.7)	255 (63.3)	960 (57.8)	0.018 *
	No or sometimes	42 (32.3)	148 (36.7)	702 (42.2)	
Overall KAP score	High	97 (74.6)	285 (70.7)	1068 (64.3)	0.005 *
	Low	33 (25.4)	118 (29.3)	594 (35.7)	

Group 1: parents of children with chronic diseases on long-term medications. Group 2: parents of healthy children on long-term medications. Group 3: parents of healthy children who are not on long-term medications. IDK: I don’t know. * Statistically significant at 0.05 level (chi-square test).

**Table 4:** Multiple logistic regression analysis of sociodemographic characteristics and risk factors associated with adequate knowledge, positive attitude, and practices related to pediatric oral medications.

		Adjusted OR	95% CI	*p*-Value
Group	Group 1	1.60	1.06–2.42	0.026 *
	Group 2	1.35	1.06–1.72	0.014 *
	Group 3	Reference		
Mother age	≤30 years	1.19	0.83–1.71	0.333
	31–40 years	1.30	0.99–1.71	0.060
	41 years or more	Reference		
Father age	≤40 years	0.76	0.59–0.99	0.039 *
	41 years or more	Reference		
Mother education	High school or less	0.66	0.48–0.91	0.011 *
	Diploma or University	0.73	0.57–0.922	0.009 *
	Higher Education	Reference		
Father education	High school or less	1.26	0.92–1.72	0.149
	Diploma or University	0.99	0.78–1.24	0.915
	Higher Education	Reference		
Family income	<10,000 SAR	0.99	0.76–1.31	0.990
	10,001–20,000 SAR	0.82	0.64–1.04	0.098
	>20,000 SAR	Reference		
Number of children less than 18 years	1	0.82	0.61–1.11	0.191
	2–3	1.09	0.85–1.40	0.475
	4 or more	Reference		

Group 1: parents of children with chronic diseases on long-term medications. Group 2: parents of healthy children on long-term medications. Group 3: parents of healthy children who are not on long-term medications. * Statistically significant at 0.05 level.

## Data Availability

The data presented in this study are available on request from the corresponding author.

## Abbreviations

The following abbreviations are used in this manuscript:

POMs	Pediatric oral medications
KAP	Knowledge, attitude and practices
CVI	Content Validity Index
CI	Confidence intervals
OR	Odds ratios

## Appendix A

Dear parent,								
We’d like to invite you to take part in our online survey, which aims to evaluate parents’ awareness, attitudes, and behaviors regarding pediatric oral medications and their potential to cause tooth decay in Saudi Arabia.								
This study is being conducted by a pediatric master’s student in the Department of Pediatric Dentistry under the supervision of a research team at the faculty of Dentistry, King Abdulaziz University.								
Please fill out the questionnaire if your children are between 2 and 18 years old.								
The survey will take 6–8 min. All survey information will be kept strictly confidential and will only be seen by the research team and will only be used for research purposes.								
Your participation in this research is voluntary and will serve the community to increase awareness in the future.								

The principal investigator,								
Department of Pediatric Dentistry								
King Abdulaziz University								
Consent Page:								
Did you read and understood the part about participants’ information in the current study?								
Yes								
No								
Do you know that you participation is voluntary and that you are free to decline at any time?								
Yes								
No								
Do you agree to participate in this survey?								
Yes								
No								
General information about the family:								
Q1: What is the marital status of the child’s parents?								
		Married	Divorced		Widowed	Deceased		
Mother’s marital status								
Father’s marital status								
Q2: What are the ages of the child’s parents?								
		<30 years	31–40 years		41–50 years	>50 years		
Mother’s age								
Father’s age								
Q3: What is the educational level of the child’s parents?								
		<High school	High school		Diploma or University	Master/PhD		
Mother’s educational level								
Father’s educational level								
Q4: What is the profession of the mother?								
(If the options do not apply, please write the exact position in the (other) field)								
- Housewife - An employee outside the health sector - An employee in the health sector in the dental field - An employee in the health sector, but not in the dental field - Self-employed person - Retired - Other								
Q5: What is the profession of the mother?								
(If the options do not apply, please write the exact position in the (other) field)								
- Unemployed - Employee outside the health sector - An employee in the health sector in the dental field - An employee in the health sector, but not in the dental field - Self-employed person - Retired - Other								
Q6: How many children are in the family (18 or younger)?								
- _______								
Q7: With whom does the child live?								
- Mother - Father - Mother and father together - Other								
Q8: What is the monthly household income?								
- <(5000) SAR - (5000–10,000) SAR - (10,001–20,000) SAR - (20,001–50,000) SAR - >(50,000) SAR								
Q9: What kind of housing do you live in?								
- Rental apartment - Owned apartment - Rental villa - Owned villa - Other								
Q10: What is the region that you live in?								
- Riyadh - Mecca - Medina - Qassim - Eastern Province - Asir - Tabuk - Hail - Northern B orders - Jizan - Najran - Al-Baha - Al-Jawf								
Knowledge, Attitude and Practices:								
In this section, we would like to explore the extent of your knowledge, attitude, and practices towards children’s oral medications in their various forms (syrup-chewable-pills-inhaler) and their impact on oral and dental health								
Q11: Children’s oral medications or some of them contain sugar flavorings.								
- True - False - I don’t know								
If the participant chooses (True) will be directed to this following question,								
Q11a: Which forms of the following oral medications may contain sugar flavorings? (Please select all possible options)								
- Syrup - Inhaler - Pills - Chewing								
Q12: Do some children’s oral medications increase the possibility of tooth decay?								
- Yes - No - I don’t know								
Q13: Do some children’s oral medications lead to tooth erosion?								
- Yes - No - I don’t know								
Q14: The aim of adding sugary flavorings in oral medications is to improve the health of the child?								
- True - False - I don’t know								
Q15: Some children’s oral medications lead to dry mouth.								
- True - False - I don’t know								
If the participant chooses (True) will be directed to this following question,								
Q15a: Which forms of the following oral medications may lead to dry mouth?								
(Please select all possible options)								
- Syrup - Inhaler - Pills - Chewing								
Q16: I think it is important to preserve the baby’s milk teeth.								
- Agree - Neutral - Disagree - I don’t know								
Q17: I think that decay of the baby’s teeth may affect permanent teeth.								
- Agree - Neutral - Disagree - I don’t know								
Q18: I think it is important to brush the teeth daily								
- Agree - Neutral - Disagree - I don’t know								
Q19: What do you usually ask your child/children to do after taking an oral medication?								
- Drinking water - Rinsing their mouth, - Brushing their teeth Nothing								
If the participant chooses (Drinking water, rinsing their mouth) will be directed to this following question,								
Q19a: If your child rinses/drinks some water after taking oral medications, please state the reason								
- ______________________________________________								
Q20: It is important for your child to drink water or rinse his or her mouth after taking an oral medication.								
- Agree - Neutral - Disagree - I don’t know								
Q21: Do you take your children to the dentist on a regular basis, at least once or twice a year?								
- Yes - No								
Q22: Do your children brush their teeth twice a day on a regular basis?								
- Yes - No - To some extent								
Q23: Daily brushing of teeth is done with water only								
- True - False - I don’t know								
Q24: Do you read the ingredients on the outside packet or the attached sheet inside the box before using any medications?								
- Yes - No - Sometimes								
Q25: Do you have any children who used to take drugs by mouth for at least 3 months, whether on prescription or without a prescription (for example, vitamins)?								
- Yes - No								
If the answer is (yes), move on to the next section								
Questions for healthy children on long term medications or medically compromised on long term medications								
Please choose one child from the family who is between the ages of 2 and 12 years old and has been taking any type of oral medication or vitamin for at least 3 months and answer the following questions:								
Q26: What is the child’s order in the family?								
- First - Middle - Last								
Q27: Does the child suffer from any chronic diseases?								
- Yes - No								
Q28: What kind of medications does your child take?								
- Syrup - Pills - Chewing - Inhaler - Other								
Q29: Your child is usually given medications								
	Once		Twice	Three times	4 or more		When needed	
When needed								
Daily								
Weekly								
Monthly								
Q30: How long has the child been taking/has been taking the medication?								
- 3–6 months - 6–12 months - >12 months								
Q31: When time of the day does your child take his/her medications?								
(You can choose more than one answer)								
- Morning - After meals - Evening - at sleep - Other								
This questionnaire has been filled out by:								
- Mother - Father - Brother/Sister - Grandparent - Others								
